# Five‐Year (2017–2022) Evolutionary Dynamics of Human Coronavirus OC43 in Southern France Based on Whole Genome Next‐Generation Sequencing

**DOI:** 10.1002/jmv.70726

**Published:** 2025-12-03

**Authors:** Hikmat Houmadi, Céline Boschi, Marine Lefebvre, Lorlane Le Targa, Justine Py, Jean‐Christophe Lagier, Lucile Lesage, Jacques Fantini, Aurélie Morand, Bernard La Scola, Philippe Colson

**Affiliations:** ^1^ Microbes Evolution Phylogeny and Infections (MEPHI) Aix‐Marseille University (AMU) Marseille France; ^2^ IHU Méditerranée Infection Marseille France; ^3^ Assistance Publique‐Hôpitaux de Marseille (AP‐HM) Marseille France; ^4^ INSERM UA 16 Aix‐Marseille University Marseille France; ^5^ Biosellal Lyon France; ^6^ Service de Pédiatrie Générale, Hôpital Timone, Assistance Publique‐Hôpitaux de Marseille (AP‐HM) Marseille France; ^7^ Service d'Accueil des Urgences Pédiatriques, Hôpital Nord, Assistance Publique‐Hôpitaux de Marseille (AP‐HM) Marseille France

**Keywords:** genomics, human coronavirus OC43, lineages, mutations, recombination, respiratory infections

## Abstract

HCoV‐OC43 genomes and their evolution are scarcely studied worldwide and in France, with only 361 genomes available as of October 2023. Here, an in‐house PCR amplification system was implemented to obtain retrospectively by next‐generation sequencing, then analyze HCoV‐OC43 genomes for infections diagnosed with this virus in southern France between February 2017 and October 2022. Multiplex PCR amplification using a set of in‐house primers designed with the Gemi software was carried out on residuals of HCoV‐OC43 RNA‐positive nasopharyngeal samples, before next‐generation sequencing (NGS) using Illumina technology on a NovaSeq. 6000 instrument. HCoV‐OC43 genome assembly, bioinformatic analyses, and phylogeny reconstruction were then carried out using CLC Genomics, Mafft, BioEdit, Nextstrain, Nextclade, MEGA, iTOL, RDP4, and HyPhy softwares. Spike structural predictions used AlphaFold and HyperChem. A total of 34 PCR primer pairs were designed for amplification, before NGS of amplicons generated from HCoV‐OC43 genomes. A total of 185 genomes were obtained, 17, 79, and 89 belonging to Genotypes G, J, and K, respectively. These three genotypes circulated exclusively or co‐circulated according to the year. A total of 303, 940, and 1300 amino acid substitutions were detected in genomes of genotypes G, J, and K, respectively, compared with reference genomes of the same genotype dating back to 2017–2018. Possible recombinations were detected for five HCoV‐OC43 genomes and involved genomes classified in genotypes J or K. HCoV‐OC43 genes encoding the hemagglutinin esterase, the spike, and the Nsp14 protein harbored the greatest number of sites under positive selection. Signature amino acid mutations F467V and S507G in the spike protein of genotypes J and G, respectively, were predicted to decrease the binding of 43E6 neutralizing antibody. Overall, the present study more than doubled the set of HCoV‐OC43 genomes available worldwide for the 2017–2022 period and contributed to the monitoring of the HCoV‐OC43 evolutionary dynamics.

## 1 Introduction

Coronaviruses affect both humans and other animals [1]. Human coronaviruses encompass so‐named endemic, or seasonal coronaviruses, including HCoV‐OC43, HKU1, NL63, and 229E, as well as epidemic or pandemic coronaviruses, including SARS‐CoV, MERS‐CoV, and SARS‐CoV‐2 [2, 3]. Endemic coronaviruses are most often responsible for asymptomatic infections or mild upper respiratory diseases. HCoV‐OC43 was, in several studies, the most frequently diagnosed human endemic coronavirus [4, 5, 6, 7, 8, 9]. It was reported that the virus exhibited seasonality in winter and spring in Western developed countries [10, 11]. In tropical countries, the seasonality of this virus is less clear [3, 12, 13].

HCoV‐OC43 was one of the two first discovered human coronaviruses, in England during the 1960s, and was first isolated from an organic tissue culture [14]. Natural and intermediate hosts for HCoV‐OC43 were described to be mice and cattle, respectively [15]. This viral species was reported to share a common ancestor with a bovine coronavirus, and a bovine‐to‐human spillover was suspected to have occurred during the late 19th century [15] while the most recent common ancestor of all genotypes was proposed to date back to the 1950s [16]. HCoV‐OC43 belongs to the genus *Betacoronavirus* [15]. Its genome is approximately 30 kilobases in length, similar to that of the other coronavirus genomes. Two‐thirds of this genome encode nonstructural (Ns) proteins (Nsp1‐16) and the remaining part encodes structural and accessory proteins. The HCoV‐OC43 genome notably encodes a haemagglutinin esterase (HE), and accessory proteins include Ns2 and Ns12.9 [17]. HCoV‐OC43 was reported to use 9‐O‐acetylated sialic acids as a receptor to bind to host cells [18, 19].

Since the advent of next‐generation sequencing (NGS), genomic data and studies have particularly expanded for some viruses, but others have remained neglected so far. Thus, while there are currently over 8 million SARS‐CoV‐2 genomes in GenBank (https://www.ncbi.nlm.nih.gov/genbank/) [20] and 17 million in Gisaid (https://gisaid.org/) [21], only 361 (near) full‐length HCoV‐OC43 genomes (with a minimum coverage of 77%, that is, a minimum size of 28 861 nucleotides) were available in GenBank as of October 2023. Moreover, only a few studies were carried out on the genomic monitoring of this virus on large sample sets. Eleven Genotypes (A–K) and an emerging lineage were described based on studies that analyzed genomes or the spike‐encoding gene (S) [7, 13]. Here, the evolutionary dynamics of HCoV‐OC43 infections diagnosed between 2017 and 2022 in Southern France was retrospectively studied, based on viral genomes obtained by NGS of overlapping DNA amplicons generated using in‐house PCR amplification systems.

## 2 Materials and Methods

### 2.1 Clinical Samples

NGS of HCoV‐OC43 genomes was performed retrospectively from remains of nasopharyngeal samples that had been diagnosed as HCoV‐OC43 RNA‐positive by real‐time reverse‐transcription (RT)‐PCR (qPCR) using the FTD Respiratory pathogens 21 assay (FTD) (Fast Track Diagnosis, Luxembourg) after extraction by the KingFisher Flex system (Thermo Fisher Scientific, Waltham, MA, USA), or by the FilmArray Respiratory panel 2 plus assay (BioFire) (Biomérieux, Marcy‐l'Etoile, France), according to the manufacturer's protocols. These clinical samples had been sent to the clinical microbiology laboratory at the University and Public Hospitals of Marseille, Southeastern France, for the purpose of routine diagnosis of respiratory infections in the setting of clinical routine management; they had been stored at −20°C or −80°C after their processing. A total of 1007 nasopharyngeal samples were diagnosed as HCoV‐OC43‐positive between February 2017 and October 2022 (69 months), and for 434 of them, a sufficient volume remained on which the PCR amplification and NGS procedures could be applied.

### 2.2 Primer Design for PCR Amplification of Overlaping Regions Covering the Whole Viral Genome

To design PCR primers, 220 complete HCoV‐OC43 genomes were collected from the NCBI GenBank (https://www.ncbi.nlm.nih.gov/genbank/) and ViPR (https://www.bv-brc.org/view/Virus/10239) sequence databases. Then, these genomes were aligned using the Mafft (Multiple Alignment using Fast Fourier Transform) software [22], and the primers were designed using the GEMI software [23], an automated tool that enables quick and easy primer designs based on nucleotide conservation in a sequence alignment. GEMI was run by setting up amplicon size (500–2600 nucleotides), amplicon overlap size ( ≥ 20 nucleotides), primer size (17–23 nucleotides), hybridization temperature (58°C–65°C), and also the maximal number of degenerated nucleotide positions ( ≤ 3). Primer specificity was then tested *in silico* using the NCBI BLAST tool (https://blast.ncbi.nlm.nih.gov/Blast.cgi) [24]. After primers were tested in simplex PCR assays and PCR were optimized using HCoV‐OC43 RNA‐positive cell culture supernatants, two sets of PCR primers were chosen, separating primer pairs into two pools to prevent the overlap of amplicons.

### 2.3 PCR Amplification of Overlaping Regions Covering the Whole Genomes

Extracted RNA was amplified by standard RT‐PCR using the SuperScript III One‐Step RT‐PCR Kit with Platinum Taq High Fidelity (Invitrogen, Life Technologies, Carlsbad, USA) according to the following protocol: 3 µL of RNA were added to 12.5 µL of 2× mix, 0.7 µL of polymerase, 1 µL of each primer pool, and 7.8 µL of pure water for a final volume of 25 µL. The PCR consisted of an initial RT step for 25 min at 50°C, followed by an initial denaturation step for 2 min at 95°C, and by 40 PCR cycles including each 15 s at 95°C, 45 s at 58°C, and 150 s at 70°C; a final elongation step consisted of 5 min at 70°C. Then, PCR amplicons were purified using the NucleoFast 96 PCR kit (Macherey Nagel, Hoerdt, France) for an elution volume of 40 µL. Eventually, PCR amplicons from the two PCR pools were mixed together as follows: 15 µL of Pool No. 1 and 25 µL of Pool No. 2.

### 2.4 Next‐Generation Sequencing

To test the PCR primers for the multiplex amplification system separately and pooled on HCoV‐OC43 RNA‐positive cell culture supernatants, the NGS was performed using the Oxford Nanopore Technology with the Ligation Sequencing Kit (SQK‐LSK109) then library sequencing on a GridION instrument after deposit on a SpotON flow cell Mk I, R9.4.1, following to the manufacturer's instructions (Oxford Nanopore Technologies, Oxford, UK). Thereafter, when the “Artic‐like” system was deemed efficient in obtaining a near full‐length genome, the NGS was performed on RNA extracts obtained from remains available for nasopharyngeal samples that had been diagnosed as HCoV‐OC43 RNA‐positive in the laboratory, using the Illumina technology on a NovaSeq. 6000 instrument. At this stage, NGS used the COVIDSeq protocol (Illumina Inc., San Diego, CA, USA), but by replacing the COVID‐19 ARTIC PCR primers with PCR primers designed here, and using the conditions previously set up. A classic loading procedure on a SP flow cell was carried out according to the NovaSeq‐XP workflow and a previously described procedure [25], with a reading of 2 × 50 nucleotides.

### 2.5 Bioinformatic Analyses

A workflow was developed using the CLC genomics workbench software v.7, consisting of a quality control step that included a trimming with a minimum of 15 nucleotides in the reads, followed by consensus genome generation by a mapping step of NGS reads against a reference genome using as parameters a coverage ≥ 80% and a nucleotide identity ≥ 90%. For the phylogenetic analysis, consensus genome sequences were aligned using the Mafft software [22] with default parameters, then the tree was built using Iqtree2 [26] with the maximum likelihood method and 1000 bootstrap replicates, and it was visualized and annotated using the iTOL software [27]. To search for amino acid and nucleotide mutations, the Nextclade and Nextstrain tools (https://nextstrain.org) [28] were modified to be used for HCoV‐OC43 sequences, and analyses were conducted separately according to the genotype. Reference genomes GenBank accession no. MN026164.1 (dating back to 2018) for genotype G, OK318939.1 (dating back to 2018) for genotype J, and MW532118 (dating back to 2017) for genotype K were used. Viral genes were classified into “structural,” “informational,” “other nonstructural,” and “accessory” categories as in a previous study [29]; the HE encoding gene and the N2 part of the nucleocapsid encoding gene were added in the structural gene category, while the category of accessory genes included Ns2 and Ns12.9. Possible recombination events into HCoV‐OCV43 genomes were searched out using the RDP4.101 software [30] on genomes with at least 95% coverage of reference genomes. This software concurrently performs seven tests, including RDP, Geneconv, Bootscan, Maxchi, Chimaera, SiSscan, and 3Seq. A potential recombination was defined here by 7 positive tests with a *p* value ≤ 0.001, to ensure a robust detection of recombinant genomes. Finally, to explore the selection pressures acting on viral genes, positive (diversifying) and negative (purifying) selection analyses were performed on structural and informational genes using the Mixed Effects Model of Evolution (MEME) that detects both episodic and pervasive positive selection [31] and the Fixed Effects Likelihood (FEL) model [32] that uses a maximum‐likelihood approach to infer nonsynonymous and synonymous substitution rates; both models were available in the HyPhy v2.5 software [33], then visualized using the web application (http://vision.hyphy.org/). Positively selected sites were identified according to the default thresholds with *p* values of 0.1 for MEME and ≤ 0.1 for FEL.

### 2.6 Analysis of the Structure of the HCoV‐OC43 Spike and of the Impact of Mutations on the Recognition by Neutralizing Antibodies

The structural study was carried out in silico from the PDB (https://www.rcsb.org/) file 7PNQ [34] of the HCoV‐OC43 spike protein and using amino acid sequences obtained here for the three genotypes G, J, and K from this protein. To fill the sequence gaps in the PDB file, the AlphaFold algorithm (https://alphafoldserver.com/) [35] was used and gave acceptable results for the domain of interest, that is, the neutralization epitope between amino acids 303 and 663. Nevertheless, this structure obtained by AlphaFold was minimized by the Polak–Ribière algorithm of the HyperChem software according to previously described conditions [36]. In a second step, this AlphaFold structure was introduced into the 7PNQ file to reconstruct a model of interaction of the HCoV‐OC43 spike protein with the neutralizing antibody 43E6. The impact of the 497–508 loop, which was not structurally resolved but present in the model used in this study, could thus be verified. This complex was used as a reference to analyze the mutations of interest in genotypes G, J, and K and their impact on antibody recognition [37]. The structure of the HCoV‐OC43 spike protein bound to its sialic acid receptor was retrieved from PDB file 6OHW [38].

## 3 Results

### 3.1 Primer Design for PCR Amplification of Overlaping Regions Covering the Whole Genomes

Of a total of 220 HCoV‐OC43 genomes collected from GenBank, 165 were selected for PCR primer design. A total of 200 PCR primer pairs were obtained by GEMI, and 34 were eventually retained. These PCR primers had a maximum of two degenerated nucleotides, their size varied between 17 and 23 nucleotides, and the expected sizes of the amplicons varied between 500 and 2600 nucleotides. Melting temperatures varied between 58°C and 65°C, and the PCR amplicons overlapped by at least 20 nucleotides. These 34 primer pairs were tested individually, and their efficacy was verified by visualizing the presence of bands on a 1.5% agarose gel after DNA staining and electrophoretic migration. PCR optimization was carried out by increasing the primer concentration in case of weak bands on the agarose gel. Eventually, the 34 PCR primer pairs were mixed into two different pools of 17 primer pairs each to avoid overlaps between the PCR amplicons, with primer concentrations ranging between 10 and 15.3 µM (Table 1).

**Table 1:** Sequences of primers used in the multiplex PCR amplification system and conditions of use.

IName	Sequence (5′–3′ orientation)	Pool no.	Concentration for use (pmol/µL)
OC43_F1	TAGGCAGTG**R**CCC**D**CCCA	1	15.3
OC43_R1	GTCTTCAGTAGG**R**TTAGCAACAT		
OC43_F2	GGCATGGCATGTTGTGCGT	2	12.6
OC43_R2	CACT**S**TCCACTACTATATAAGC		
OC43_F3	TGGA**Y**TCTTTAGGTGCAGCT	1	15.3
OC43_R3	ACTTCAAC**Y**ACACTATC**Y**GCT		
OC43_F4	GCCTAGTCAAGTTCAGAAAGC	2	10.0
OC43_R4	CAGA**K**GT**M**TCCACACTTGGAC		
OC43_F5_alt2	TCCAAGTGTGGA**K**AC**M**TCTGA	1	15.3
OC43_R5_alt2	TTAACATCCACAACACAAGCTG		
OC43_F6	GTTGCTGATTTTGCATATTGGT	2	10.0
OC43_R6	TCCAACAACATTAAGCACAGT		
OC43_F7_alt1	GCAGTAGCAGCTGGACAG	1	15.3
OC43_R7_alt1	TCTT**R**CCAAAACT**W**TCACCAAC		
OC43_F8_alt2	TGAGTGTACTGGAGGCATAG	2	10.0
OC43_R8_alt2	TACAACCCCTCTCATATCTCT		
OC43_F9_alt1	GTGGCCAACAGCTACAGG	1	10.0
OC43_R9_alt1	CCTGCCA**R**GCAGAACTGAC		
OC43_F10	GGTTGCTAGAGG**Y**GCTTGC	2	10.0
OC43_R10	AACATGCATATTAGGCACACTCT		
OC43_F11	C**Y**ACTTGGCTGCC**W**GAGC	1	15.3
OC43_R11	TCATCCGTCAATTC**R**A**B**ACCTG		
OC43_F12_alt1	TGCTTTAATAGCAACTGCGCA	2	10.0
OC43_R12_alt1	CCACAAAAGGTCCCAGGC		
OC43_F13	GTGAGGAAGCTGATGAGGG	1	15.3
OC43_R13	GAACATAT**Y**AC**R**TGTCTGGGAC		
OC43_F14	ACTGTCCCAGACA**Y**GT**R**ATATG	2	15.3
OC43_R14	AGTATAGCAGCATA**R**AGCCATG		
OC43_F15_alt1	TAGTACTGGTTGTCATAC**W**GGT	1	15.3
OC43_R15_alt1	ACTGAGCAAGCTTTTCAAAAGC		
OC43_F16	GCCACTTCGGATCTGAGTG	2	10.0
OC43_R16	GTGGT**R**GCATCGGGTTTAACA		
OC43_F17	GC**Y**GGTAC**Y**GGTATGGCC	1	10.0
OC43_R17	TACACGCTCATAGCATTTCATC		
OC43_F18_alt1	GCCCGTCTCGTACCCTG	2	10.0
OC43_R18_alt1	CTAAAACAGCAAGTGCGTAAAT		
OC43_F19	GC**W**GCTGATCCAGCTTTGC	1	10.0
OC43_R19	TAGCAAAAGCAGTAGTTGCATC		
OC43_F20	AAATTGT**Y**ATGTGTGGTGGCTGT	2	15.3
OC43_R20	CATCATTTACATCACATCCTGG		
OC43_F21	GGCGACTGATCATAAATATGTC	1	15.3
OC43_R21	ACATTGTTCTGAAACGTCTC**R**AG		
OC43_F22_alt1	GC**R**TCAGCAACAATACAAGAG	2	10.0
OC43_R22_alt1	ATCTGCTGCATATTTACAGCAC		
OC43_F23_alt2	ACAGT**K**TC**Y**GCCTTGGTTTAT	1	15.3
OC43_R23_alt2	ATGGCGCCAACAACCATA**R**TA		
OC43_F24	GGGCAGCCAACTTTGAGC	2	15.3
OC43_R24	ACCTGCTTAGCATCCAT**R**CC		
OC43_F25	TTGAG**Y**ATTTGAAGCCTATGCCA	1	15.3
OC43_R25	CCCCAGATTACCTTGTGGG		
OC43_F26_alt2	CAGCTCTAACCAGAGCCCA	2	15.3
OC43_R26_alt2	TGGCATGCATAACATTTCCATC		
OC43_F27_alt2	AAGTT**R**GCTCT**D**GGTGGCAG	1	15.3
OC43_R27_alt2	ATCATCATTCTC**R**GGAAGATC		
OC43_F28_alt1	GCTCGGTGCTGAAAAG**R**AAG	2	15.3
OC43_R28_alt1	GCTAGT**R**AAACCAGCATC**Y**C		
OC43_F29_alt1	CGGTGGAACAATGC**Y**AGGC	1	15.3
OC43_R30/31_alt1	AGTTAAT**R**GGTTGCAGCTGTC		
OC43_F32_alt2	CGACAGCTGCAACC**Y**ATTAA	2	15.3
OC43_R32_alt2	AGCACATCCAT**R**G**W**GACACC		
OC43_F33	GGCTGCCAC**Y**TCTGCTAG	1	15.3
OC43_R33	CATAGCTACACCA**R**CAAGG**S**		
OC43_F34	ATGGCCTTGGTATGTATGGC	2	10.0
OC43_R34	GGGCCACAT**R**A**K**CCACAAAATA		
OC43_F35	GCAAT**W**TGG**R**TATACAAGTCGC	1	10.0
OC43_R35	AGCAGACCTTCCTGAGCC		
OC43_F36_alt1	ATCTGGGAACMGGACCGC	2	10.0
OC43_R36_alt1	GCTCRTCCATCTTCTTAAGAA		

*Note:* Degenerate nucleotide positions are indicated by bold font.

### 3.2 Next‐Generation Sequencing

After amplification of HCoV‐OC43 genomes using our in‐house multiplex PCR system, NGS was performed on 434 HCoV‐OC43 RNA‐positive nasopharyngeal samples with the Illumina technology on a NovaSeq. 6000 instrument. A total of 185 HCoV‐OC43 genomes were recovered according to the selected parameters, including a minimum coverage of 80% of full‐length reference genomes and a minimum of 3× as NGS depth relative to these reference genomes. The 185 nasopharyngeal samples from which they were obtained had been collected between February 6, 2017 and July 16, 2022 (over a period of 66 months). The qPCR cycle threshold values (Ct) were available for 96 samples and ranged between 11 and 25, with a mean (± standard deviation) of 17.8 ± 3.3 Ct. The total number of NGS reads per sample that were mapped onto the reference genome ranged between 5675 and 9 917 033, with a mean of 3 569 789 ± 2 943 862 reads, and mean coverage was of 94.7 ± 5.2%.

### 3.3 Phylogeny Reconstruction and Temporal Distribution of Genotypes

**Figure 1:**
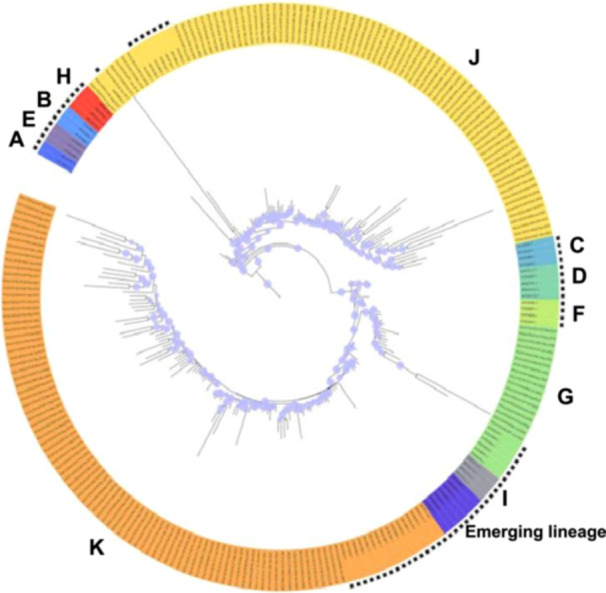
Phylogenetic tree based on HCoV‐OC43 genomes obtained in the present study and from GenBank. Bootstrap values ≥ 70% were indicated at nodes on the tree by a light blue circle. Genomes recovered from GenBank (https://www.ncbi.nlm.nih.gov/genbank/) [20] and not obtained here are indicated by a black asterisk.

**Figure 2:**
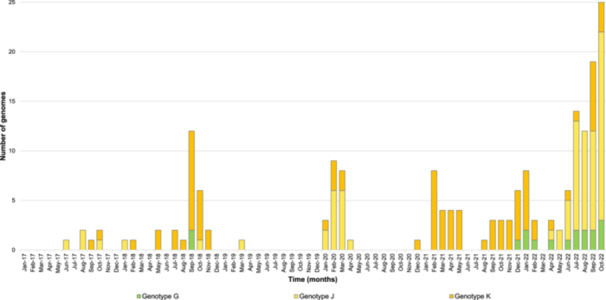
Temporal distribution of genotypes for HCoV‐OC43 genomes obtained in the present study.

Phylogeny reconstruction included HCoV‐OC43 genome sequences obtained here as well as genomes of the different genotypes from datasets obtained in previous studies [7]. Only bootstrap values ≥ 70% were displayed on the tree. Viral genomes recovered here were clustered into three genotypes, namely, G, J, and K, with 17, 79, and 89 sequences, respectively (Figure 1). Viral genotype assignment was performed based on significant clustering (bootstrap values ≥ 70%) with previously classified genomes in the phylogenetic tree, and independently using the Nextclade tool [28]. Mean nucleotide diversity between sequences obtained here was 91.9 ± 6.3% for genotype G, 93.2 ± 5.1% for genotype J, and 91.8 ± 5.3% for genotype K. Regarding the temporal distribution of the incidence of the different genotypes (Figure 2), a single genotype circulated during some periods of time, such as for the case of genotype K between November 2020 and October 2021 (Figure 2). In contrast, during other periods, several genotypes co‐circulated, as for the case with genotypes J and K in 2020, or all three genotypes G, J, and K in 2018 and in 2022.

### 3.4 Mutation Patterns

**Figure 3:**
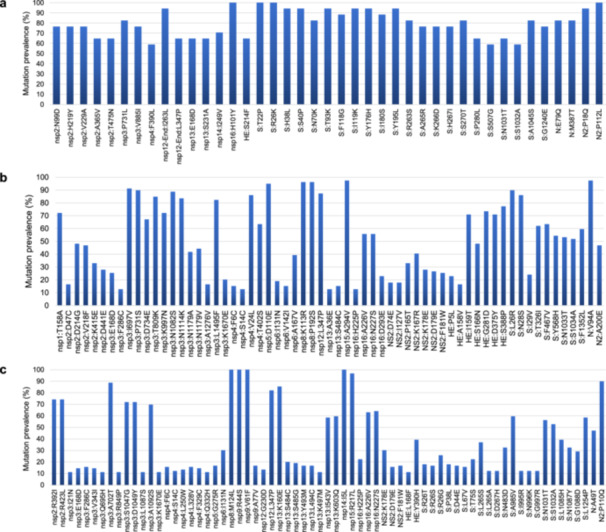
Distribution along HCoV‐OC43 genomes of the three Genotypes G (a), J (b), and K (c) obtained here of the prevalence of amino acid substitutions relative to reference genomes. References genomes were genomes GenBank (https://www.ncbi.nlm.nih.gov/genbank/) [20], Accession no. MN026164.1, OK318939.1, and MW532118.1 for Genotypes G, J, and K, respectively. HE, hemagglutinine esterase; N, nucleocapsid; NS, nonstructural; nsp, nonstructural protein; S, spike.

The patterns of mutations within genomes were analyzed for each viral genotype separately. Regarding genotype G genomes, 919 nucleotide substitutions were detected compared with the reference genome GenBank accession no. MN026164.1 dating back to 2018. To gain a better picture of the distribution of these mutations, the genes were classified into “structural,” “informational,” “other nonstructural,” and “accessory” categories. In the informational gene category, Nsp13, which encodes the helicase, was the most affected gene, with 46 substitutions per 1000 nucleotides, and Nsp15, which encodes an endoribonuclease, was the least affected, with five substitutions per 1000 nucleotides (Table 2a). In the structural gene category, the envelope gene showed the greatest diversity with 106 substitutions per 1000 nucleotides, and the matrix encoding gene was the least affected with four substitutions per 1000 nucleotides. A total of 303 amino acid substitutions were detected relative to the reference genome MN026164.1 (Table 2a; Figure 3a). In the informational gene category, Nsp13 was the most affected with 44 amino acid substitutions per 1000 nucleotides, and Nsp8 exhibited no amino acid substitutions. In the structural gene category, the most affected gene was the envelope gene, and the least affected was the matrix gene. Forty‐one different amino acid substitutions were encoded by ≥ 10 of the genotype G genomes. The genes harboring these mutations were Nsp2, Nsp3, Nsp4, Nsp12, Nsp13, Nsp14, and Nsp16 regarding nonstructural genes, and HE, S, N, and N2 regarding structural genes. A majority of these mutations were located in the spike gene and were present in the majority of the genomes analyzed here. One mutation (S507G) was located in the receptor binding domain (RBD) of the spike protein that was predicted to span amino acids 339–549 [16]. The other genes, including Nsp1, Nsp5, Nsp6, Nsp7, Nsp8, Nsp9, Nsp10, Nsp11, Nsp15, M, E, and the accessory genes, were considerably conserved.

**Table 2:** Frequency of nucleotide and amino acid mutations in HCoV‐OC43 genotypes G (a), J (b), and K (c).

Gene	Size (nucleotides)	Number of mutations per gene	Number of mutations per 1000 nucleotides	Size (amino acids)	Number of mutations per protein	Number of mutations per 1000 amino acids
(a)						
Nsp1	738	1	1	222	1	4
Nsp2	1815	71	39	587	15	25
Nsp3	5697	130	23	2029	36	17
Nsp4	1488	107	72	496	35	70
Nsp5	909	8	9	303	2	6
Nsp6	861	4	4	287	4	14
Nsp7	267	2	7	92	1	11
Nsp8	591	4	6	194	0	0
Nsp9	330	3	9	110	1	9
Nsp10	411	5	12	137	1	7
Nsp11	42	0	0	14	0	0
Nsp12	2783	82	29	928	35	37
Nsp13	1809	85	46	603	27	44
Nsp14	1563	13	8	521	2	4
Nsp15	1125	6	5	374	4	10
Nsp16	897	22	24	299	12	40
NS2	837	65	77	278	25	90
HE	1284	58	45	427	17	40
S	4077	176	43	1358	57	42
NS12.9	330	6	18	109	2	18
E	255	27	106	84	12	143
M	693	3	4	230	1	4
N	1347	36	26	448	8	18
N2	348	5	14	115	5	43
(b)						
Nsp1	738	101	137	222	28	126
Nsp2	1815	157	86	587	58	99
Nsp3	5697	329	57	2029	107	52
Nsp4	1488	168	113	496	58	117
Nsp5	909	31	34	303	9	29
Nsp6	861	70	81	287	25	87
Nsp7	267	10	37	92	4	43
Nsp8	591	11	18	194	4	20
Nsp9	330	5	15	110	4	36
Nsp10	411	4	9	137	0	0
Nsp11	42	0	0	14	0	0
Nsp12	2783	244	87	928	47	50
Nsp13	1809	210	116	603	54	89
Nsp14	1563	45	28	521	8	15
Nsp15	1125	61	54	374	18	48
Nsp16	897	57	63	299	20	67
NS2	837	289	345	278	115	413
HE	1272	313	246	423	127	300
S	4083	713	174	1360	189	139
NS12.9	330	11	33	109	3	27
E	267	12	45	88	7	79
M	693	48	69	230	18	78
N	1344	74	55	447	22	49
N2	624	36	57	207	15	72
(c)						
Nsp1	738	64	86	222	14	63
Nsp2	1815	246	135	587	82	139
Nsp3	5697	672	118	2029	182	89
Nsp4	1488	313	210	496	106	213
Nsp5	909	105	115	303	34	112
Nsp6	861	85	98	287	25	87
Nsp7	267	11	41	92	6	65
Nsp8	591	46	78	194	22	113
Nsp9	330	21	63	110	10	91
Nsp10	411	13	31	137	5	36
Nsp11	42	0	0	14	0	0
Nsp12	2783	315	113	928	58	62
Nsp13	1809	300	166	603	78	129
Nsp14	1563	73	46	521	32	61
Nsp15	1125	78	69	374	25	67
Nsp16	897	103	115	299	39	130
NS2	837	381	455	278	138	496
HE	1284	318	247	427	114	267
S	4077	833	204	1358	228	168
NS12.9	330	31	94	109	11	101
E	255	49	192	84	15	178
M	693	78	112	230	20	87
N	1347	117	87	448	38	85
N2	624	57	91	207	18	87

Abbreviations: HE, hemagglutinine esterase; N, nucleocapsid; NS, nonstructural; nsp, nonstructural protein; S, spike.

Regarding genotype J genomes, their mutation patterns were analyzed relative to the reference genome OK318939.1 that dates back to 2018. In the informational gene category, the gene with the greatest nucleotide diversity was Nsp13, and the one with the lowest diversity was Nsp10, with 116 and 9 substitutions per 1000 nucleotides, respectively (Table 2b). In the structural gene category, the gene most affected was the one encoding HE with 246 substitutions per 1000 nucleotides, followed by the spike gene with 174 substitutions per 1000 nucleotides, and the gene least affected was the envelope gene with 45 substitutions per 1000 nucleotides. A total of 940 amino acid substitutions were observed to be encoded by the genotype J genomes obtained here, relatively to genome OK318939.1 (Table 2b; Figure 3b). In the informational gene category, Nsp13 was the most mutated with 89 amino acid substitutions per 1000 nucleotides, and Nsp10 was the least mutated with no amino acid substitutions. In the informational gene category, Nsp13 was the most affected with 89 amino acid substitutions per 1000 nucleotides, and Nsp10 was the least affected with no amino acid substitutions. In the structural gene category, the HE encoding gene was the one exhibiting the greatest amino acid diversity, while the spike and nucleocapsid genes were those the less mutated. In the accessory gene category, Ns2 showed the greatest amino acid diversity with 413 amino acid substitutions per 1000 nucleotides. Overall, 64 amino acid substitutions were encoded by ≥ 10 genotype J genomes. A great number of mutations were observed in nonstructural genes, the most mutated of which was Nsp3. Structural gene products with amino acid changes were HE, spike, and nucleocapsid. One mutation (F467V) was located in the spike RBD. There were also mutations in the Ns2 accessory gene. Finally, the most conserved gene products were Nsp7, Nsp10, Nsp11, Nsp14, M, E, and the other accessory proteins apart from Ns2 (Figure 3b).

Regarding genotype K genomes, mutations were analyzed relative to genotype K reference genome MW532118.1, which dates back to 2017. The gene with the greatest nucleotide diversity in the informational gene category was Nsp13, and the gene with the lowest diversity was Nsp10, with 166 and 31 substitutions per 1000 nucleotides, respectively. In the structural gene category, the HE gene was the one with the greatest diversity with 247 substitutions per 1000 nucleotides, followed by the spike gene with 204 substitutions per 1000 nucleotides, while the nucleocapsid gene was the one with the lowest diversity with 87 substitutions per 1000 nucleotides (Table 2c). Ns2 was the accessory gene with the greatest diversity, with 455 substitutions per 1000 nucleotides. Besides, a total of 1300 amino acid substitutions were detected in the genomes obtained in the present study relative to the reference genome MW532118.1. In the informational gene category, the greatest amino acid diversity was observed for Nsp16 and Nsp13, with 130 and 129 amino acid substitutions per 1000 nucleotides, respectively. Nsp10 was the gene least affected by mutations in this category. In the structural gene category, the HE gene was the gene with the greatest diversity, and the nucleocapsid gene was the gene with the lowest diversity, with 267 and 85 amino acid substitutions per 1000 nucleotides, respectively. Ns2 was the accessory gene with the greatest diversity (Table 2c; Figure 3c). Overall, 68 amino acid changes were found in ≥ 10 genomes. They were distributed in almost every gene, in both nonstructural and structural genes, including in Nsp2, Nsp3, Nsp4, Nsp5, Nsp6, Nsp8, Nsp9, Nsp12, Nsp13, Nsp14, Nsp15, Nsp16, HE, S, N, N2, and the accessory gene Ns2. One mutation (N483D) was located in the spike RBD. The most conserved proteins were Nsp1, Nsp7, Nsp10, Nsp11, M, E, and the other accessory proteins apart from Ns2.

### 3.5 Detection of Positive and Negative Selection by HyPhy (MEME and FEL)

Detection of sites evolving under positive and negative selection was performed for structural and nonstructural genes using the MEME and FEL methods. Regarding the MEME analysis, all structural genes except the envelope gene were found to harbor positively selected sites, and this was the case for all informational genes except for Nsp7, Nsp8, and Nsp10 (Supporting Information: Table S2 and Figure S2A–O). Among structural genes, the number of sites predicted to be under positive selection was particularly high for the gene encoding the HE, a 1284 amino acid‐large protein, with 109 such sites, and also for the gene encoding the spike, a 4077 amino acid‐large protein, with 119 such sites. Regarding the FEL analysis, for structural genes, only the HE encoding gene and the spike encoding gene were found to harbor positively selected sites, while all genes harbored negatively selected sites (Supporting Information: Table S1 and Figure S2A–O). Regarding informational genes, they all harbored positively selected sites except Nsp7, Nsp8, Nsp10, and Nsp16, and they all harbored negatively selected sites. The number of positively selected sites was particularly high for the genes encoding the HE and the spike, with 97 and 33 sites, respectively, as well as for the gene encoding Nsp14, a 1563 amino acid‐large nonstructural protein, with 50 sites. Nsp14 is highly conserved in the family *Coronaviridae* [39, 40]. It is a bifunctional enzyme with an exoribonuclease domain that has a proofreading role and a S‐adenosyl methionine‐dependent (guanine‐N7) methyl transferase that facilitates viral mRNA translation and protects the virus from host innate immune defenses.

### 3.6 Genome Recombination Events

Recombination events were searched for genomes with ≥ 95% coverage using the RDP4 software. Potential recombination events were detected for genomes retrieved from five samples. Of these five genomes, two belonged to genotype J and three to genotype K (Table 3; Supporting Information: Figure S1A–E). The genotypes of major–minor parental sequences were J–K and E–K in one case each, and E–J in three cases. Therefore, these putative recombinant genomes involved a parental genome classified as of genotype E, not found in the present study. The breakpoints differed depending on the genome, as they were located in different genes. Indeed, they involved S and HE genes in two cases each, and Nsp12, Nsp13 and Nsp16, Nsp2, Nsp3, and Ns2 genes in one case each (Table 3).

**Table 3:** Putative HCoV‐OC43 recombinant genomes detected and parental genomes either obtained here or collected from GenBank.

Putative recombinant (genotype)	Minor parental genome (genotype)	Major parental genome (genotype)	Coordinates for the putative recombination regions (relatively to NC_006213.1) and genes or regions involved
HCoV‐OC43‐0059_Sep‐2022	HCoV‐OC43‐0191_Aug‐2017	HCoV‐OC43‐0033_Oct‐2022	8,170‐9,320
(K)	(J)	(K)	Nsp2‐Nsp3
HCoV‐OC43‐0191_Aug‐2017	KP198610.1	HCoV‐OC43‐0045_Oct‐2022	22,625‐25,832
(J)		(J)	HE‐S
HCoV‐OC43‐0177_Sep‐2018	KF923896.1	HCoV‐OC43‐0158_Mar‐2019	23,363‐27,379
(K)	(E)	(J)	HE‐S
HCoV‐OC43‐0058_Sep‐2022	KU131570.1	HCoV‐OC43‐0036_Oct‐2022	15,257‐16,284
(J)	(E)	(J)	Nsp12‐Nsp13
HCoV‐OC43‐0160_Nov‐2018	KU131570.1	HCoV‐OC43‐0027_Oct‐2022	21,208‐22,063
(K)	(E)	(K)	Nsp16‐NS2

Abbreviations: HE, hemagglutinin esterase; NS, nonstructural; Nsp, nonstructural protein; S, spike.

### 3.7 Analysis of the Structure of the HCoV‐OC43 Spike and of the Impact of Mutations on the Recognition by Neutralizing Antibodies

**Figure 4:**
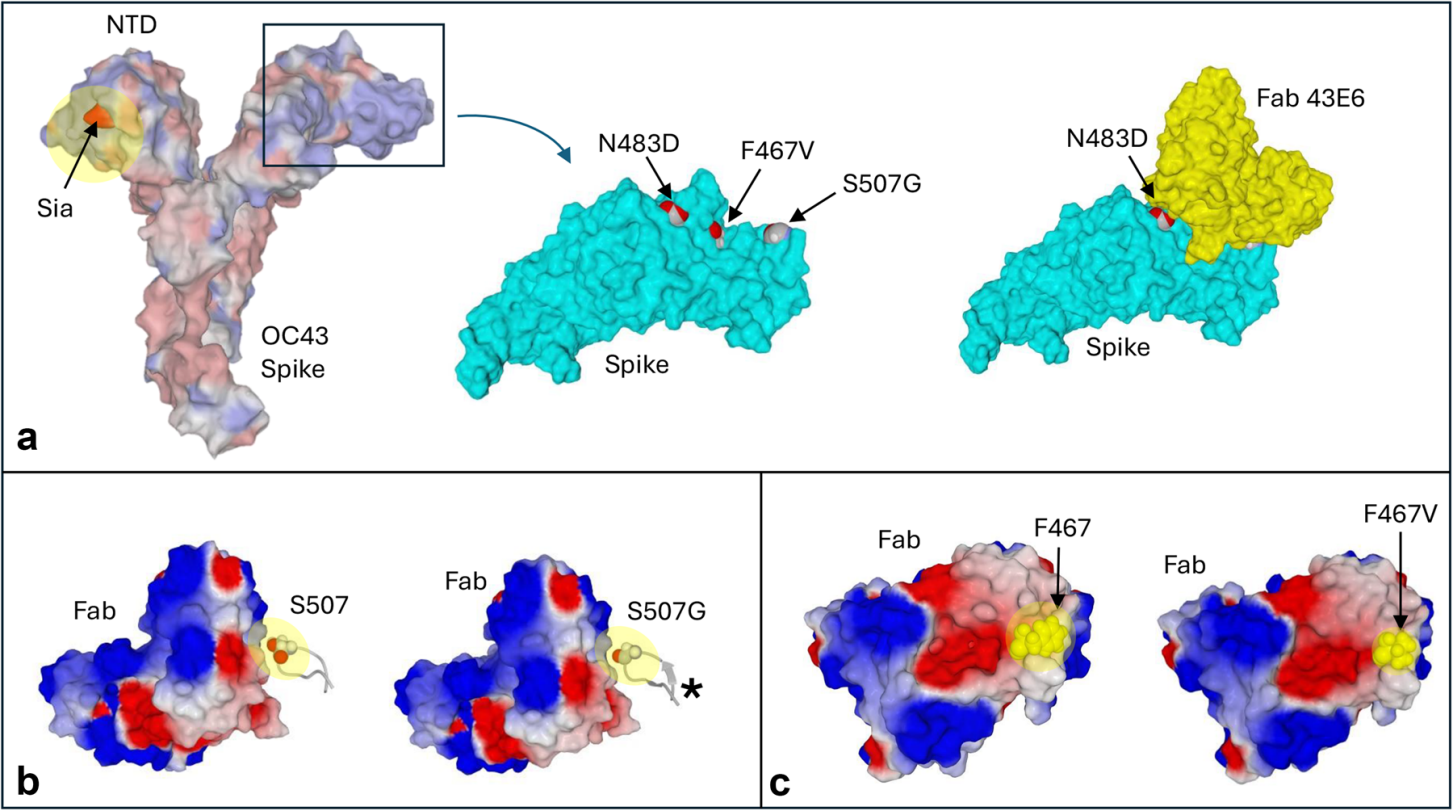
Analysis of the impact of mutations on the recognition of neutralizing antibodies. (a) In the left panel, the spike protein of coronavirus OC43 is represented in surface electrostatic potential, with the sialic acid receptor colored red. The box delimits the region of the protein containing the mutations of interest N483D, F467V, and S507G. In the middle model, the spike protein neutralization domain is shown in blue, and the three mutations are indicated. This structure has been modeled to reveal the missing loop (amino acid residues 497–508) containing position 507. The model on the right represents the complex reconstituted in this study and minimized between the corrected spike protein domain and the neutralizing antibody 43E6. Only amino acid 483 remains visible, whereas the other two positions, 467 and 507, are masked in the complex. (b) The models compare the interaction of the antibody with the spike protein presenting either S507 or the S507G mutation. The S507G mutation has a stiffening effect on the 497–508 loop (asterisk), which diminishes the fit between the protein and the antibody. (c) The models compare the interaction of the antibody with the spike protein presenting either F467 or the F467V mutation. This mutation affects the antibody binding by two mechanisms: (i) decrease in the interaction surface and (ii) less geometric complementarity between the protein and the antibody.

To assess the impact of the three signature mutations in the spike protein, namely, S507G for genotype G, F467V for genotype J, and N483D for genotype K, an in silico analysis of the corresponding spike proteins was performed. First, an interaction model of the neutralization domain recognized by the 43E6 antibody was constructed. This neutralization domain is distant from the area located in the N‐terminal domain (NTD) of the spike protein that interacts with the receptor identified for this virus, consisting of sialic acid (Figure 4a). Even if this region of the NTD is also the target of neutralizing antibodies, the mutations of interest harbored by the three viral genotypes are clearly outside this zone. In the present study, the PDB file 7PNQ, on which relied previous HCoV‐OC43 neutralization analyses [34], was taken as a reference. This PDB file, however, presented notable differences in amino acid sequence compared with sequences from the three genotypes G, J, and K considered here. Furthermore, the S507G mutation was located in an unresolved loop according to the 7PNQ PDB structure, and a complete structural model including this loop had therefore to be generated to account for this mutation. The results of this structural study are shown in Figure 4b,c. The three mutations harbored by the spike of each of the three genotypes G, J, and K are located on the protein surface at the sites of interaction with neutralizing antibody 43E6. At first glance, it seemed that each of these mutations could potentially affect the recognition by this antibody, but a more subtle prediction was inferred. Thus, at amino acid position 507 in genotype G, replacement of a serine by a glycine had a structural effect on the loop consisting in a slight loss of affinity for the antibody. Indeed, loop displays a greater flexibility when amino acid 507 is a serine, allowing fitting better with the antibody; the model in Figure 4b also shows a larger surface of interaction for S507 than for S507G. For the case of mutation F467V in genotype J, the aromatic structure of phenylalanine lies on a large flat surface, resulting in an enhanced binding capacity of the antibody. This is no longer possible when valine, a branched amino acid, replaces phenylalanine in the F467V mutant (Figure 4c). Moreover, valine significantly affects the geometric complementarity between the protein and the antibody. Finally, no major effect of the N483D mutation of genotype K on the interaction of the spike protein with the 43E6 antibody was observed. Overall, the three mutations can be classified as follows by their decreasing potential impact on the binding of the 43E6 antibody on the spike: the F467V mutation of genotype J causes a significant loss of affinity; the S507G mutation of genotype G causes a slight loss of affinity; and finally the N483D mutation of genotype K does not appear to affect on its own the binding of the neutralizing antibody. Furthermore, it was anticipated here that none of these three mutations could affect the binding of the spike protein to its sialic acid receptor, as the spike binding site is very distant from these mutations.

## 4 Discussion

As the SARS‐CoV‐2 pandemic that emerged in 2019 reminded us, coronaviruses are viruses that have a continuous potential of emergence both in nonhuman animals and in humans, and are a zoonotic source of infections for humans. Evidence of spill‐over from humans to chimpanzees of HCoV‐OC43 was also reported in the Ivory Coast [41, 42]. Therefore, it is critical to monitor the evolution of these viruses to account for the different mutants and variants observed. Among the four so‐called “endemic” coronaviruses that infect humans, HCoV‐OC43 is the one the most frequently detected. Here, 185 HCoV‐OC43 genomes were obtained and analyzed, whereas only 361 complete or near‐complete genomes were available in GenBank at the beginning of the present study (as of October 4, 2023). In addition, these GenBank sequences were obtained primarily in China and the USA, and only 11 were obtained in France. Moreover, for the 2017–2022 period studied here (as could be assessed when the respiratory sample collection date was available), there were a total of only 145 genomes available worldwide, and none were from France. Thus, the set of HCoV‐OC43 genomes available worldwide until late 2023 was expanded by approximately 50%, the set of genomes available worldwide for the 2017–2022 period was more than doubled, the number of genomes available for France was expanded by approximately 17 times, and the only genomes for the period from 2017 to 2022 of this country were provided.

PCR amplification of viral RNA pre‐NGS is often necessary, considering viral loads in clinical samples are often too low to obtain genomes, and even reads by direct NGS. This has been the case with SARS‐CoV‐2 during the pandemic with the implementation and updating of Artic systems (https://artic.network/ncov-2019/ncov2019-bioinformatics-sop.html). A protocol for whole‐genome sequencing of HCoV‐OC43 was previously described [43]. It was designed based on 47 previously available genomes and used 99 PCR primer pairs that generated 500–550 base pairs (bp) long amplicons that covered the whole genome but were pooled in 12 sets (each containing from 3 to 12 primer pairs). This protocol allowed obtaining nine full‐length or near full‐length genomes from 11 respiratory samples. Another study conducted in Russia reported in 2025 the design using the PrimalScheme application (https://primalscheme.com/) of a panel of 36 PCR primer systems generating amplicons with a size ranging between 900 and 1100 bp for HCoV‐OC43 whole genome amplification before NGS [44]. Twenty‐three genomes with > 70% coverage were obtained, with the highest coverage observed in the case of qPCR Ct of HCoV‐OC43 RNA detection < 25. Also, a study conducted in the UK reported the design and use of an amplicon‐based whole genome sequencing panel targeting all four endemic HCoV plus SARS‐CoV‐2 [45]. PCR amplicons were approximately 1200 bp‐long. A total of 128 (69%) genomes with > 95% coverage, which were of Genotypes E, G, I, J, and K, were obtained from 185 samples collected between 2016 and 2023. Here, PCR primer pairs were designed based on 220 complete genomes collected from GenBank, and 34 pairs were eventually retained that generated amplicons with sizes varying from 500 to 2600 bp and were mixed into only two pools (of 17 primer pairs each). This protocol allowed obtaining 185 full‐length or near full‐length genomes from 434 nasopharyngeal samples with various Ct values. This PCR yield was only 43% but it must be taken into account that shorter genomes, which displayed a coverage < 80% of the full‐length reference genomes, were obtained from samples from which the protocol used in this study failed to generate larger genomes. Therefore, the protocol implemented here can be distinguished by the use of a greater set of complete HCoV‐OC43 genomes than earlier to design PCR primers, which was carried out using the GEMI software [23] that automatically incorporates degenerated bases, and by the final use of only 34 pairs of primers split into only two pools. In addition, there were differences in NGS technologies and platforms used here (Illumina technology on a NovaSeq. 6000 instrument) and those used in previous studies (Nanopore technology, Illumina technology on a MiSeq instrument, or MGI technology on a DNBSEQ‐G400 instrument [MGI Tech, Shenzhen, China]). It is worthy to note that another strategy to obtain whole HCoV‐OC43 genomes in case of low viral load is an hybridization‐based enrichment with an appropriate viral panel probe set, as was for instance reported in 2018 to obtain four genomes from respiratory samples of children with severe acute respiratory infections or to investigate using genomics an outbreak in wild chimpanzees in Ivory Coast [41, 42].

The predominance observed here of genomes obtained for the 2020–2022 period is due to the COVID‐19 pandemic, which was associated with an increase in the sampling of patients with clinical symptoms of respiratory infection. Three HCoV‐OC43 genotypes were identified: G, J, and K. Genotypes J and K, which are among the latest detected HCoV‐OC43 genotypes, were reported for the first time in China in 2022 [13]. Ye et al. recently reported the detection of four genotypes (G, I, J, and K) and of an emerging lineage (branching with genotypes I and K in the phylogenetic analysis) by analyzing 179 complete or near‐complete genomes obtained from 312 HCoV‐OC43 RNA‐positive respiratory samples collected in China [7]. Three of these four genotypes also circulated in France during the period of this study, and this finding raises questions about how long they were circulating in this country. According to the period of time of this study, either a unique genotype was found to circulate (from autumn 2020 until autumn 2021) or alternatively two or three genotypes co‐circulated. Otherwise, none of the genomes obtained here belonged to the new HCoV‐OC43 lineage that was reported to have emerged in China [7].

The HE gene was the structural gene showing the greatest diversity for genotypes J and K genomes, while the envelope gene showed the greatest diversity for genotype G. The Nsp13 gene, which encodes a helicase, was the informational gene that exhibited the greatest diversity. The spike gene was one of those with the greatest diversity in the present study, which is in accordance with other studies showing that this gene evolves rapidly in coronavirus genomes [7, 16]. For instance, a considerable genetic diversity was previously reported based on clonal sequences of the S1 gene of seven clinical HCoV‐OC43 [46]. Besides, Oong et al. reported that spike mutations D267K and I268D (which correspond to D266K and I267D, respectively, in the study by Ren et al.) were signature mutations of the genotype G [47, 48]. As a matter of fact, these mutations were not carried by genotype G genomes obtained here but only by some of the genotype G genomes from GenBank. Oong et al. also reported the presence of mutations P22T and T271S in two genomes from China [48]. All the genomes obtained here carried the mutation P22T, but some genomes available in GenBank did not. As for mutation T271S, it was harbored by some but not all genotype G genomes obtained here or from GenBank. Thus, the classification of HCoV‐OC43 genomes based on signature mutations needs to be checked and refined based on genomes available at a given time period, as it depends on the number and diversity of available genomes. Notwithstanding, in the present study, some mutations were identified that were signature mutations for a genotype and persisted over the years, while others appeared over time and were markers of the virus's genetic evolution. Finally, here, one mutation located in the spike RBD, which was associated with capabilities to escape immune responses and adapt to humans [18], was identified in each of the three HCoV‐OC43 genotypes G, J, and K. Taken together, previous findings hence suggest that tracking HCoV‐OC43 evolution based on genome analysis is worthy to gain a better picture and understanding of the emergence and faith of viral lineages within and between countries. Moreover, the structural genes encoding the HE and the spike were those found here to harbor the greatest number of positions under positive selection, which is consistent with previous reports [48, 49]. Besides, the presence of negatively selected sites in all genes may indicate a conservation of key viral functions, constraining genetic variability in these regions. The number of positively selected sites was also high among nonstructural genes for the one encoding Nsp14, which was reported to have a proofreading role on the viral polymerase activity, to promote viral mRNA translation, and to interfere with the host innate immune defenses [39, 40]. Otherwise, a limitation of the present study is that it did not perform haplotype network analyses, which would be interesting avenues for future research conducted on larger sets of genomes obtained from samples collected during long periods of time.

HCoV‐OC43 is a coronavirus known to frequently recombine [16, 50, 51]. Recombination between two different “parental” genomes occurs when there is a coinfection of the same host cell, resulting in the emergence of a virus that harbors parts of the genetic material of both “parents.” Several of the 11 currently defined HCoV‐OC43 genotypes (A–K) were reported to be derived from recombination events or from a recombinant “parent.” Regarding genotype K, it was previously reported to result from a recombination involving a parental genome of genotype I [13], while Genotype J was previously reported as being involved in recombination involving parental genomes of genotypes H and I [13], and genotype H was suspected to have occurred from recombination between parental genomes of genotypes F/D‐like, E, and B [50]. In the present study, three genotypes were putatively involved in five different combinations, and putative recombinations involved eight different genes of the HCoV‐OC43 genomes. Putative recombinants involved genotypes K and J, which were those very majorities in the data set and which co‐circulated during several periods of time. Three of the five putative recombinant genomes comprised a genotype J fragment as a major or minor parental genome in three and one cases, respectively.

Finally, analysis of the structure of the HCoV‐OC43 spike protein and of the impact on antibody neutralization of three signature mutations, one for each of the three HCoV‐OC43 genotypes identified here, suggested various or no effect depending on the mutation. Thus, two of these mutations in the spike of viruses of genotypes J and G were predicted to cause a loss of affinity for a neutralizing antibody, which suggested a greater potential for immune escape for these genotypes. Interestingly, the greatest loss of affinity was observed for genotype J that became the predominant genotype during the late months of this study period. It is also worth noting that the mutations studied here were located at a substantial distance from epitopes targeted by such neutralizing antibodies as 43E6, and that none of them were predicted to affect the receptor binding site for 9‐O‐acetylated sialic acid [34].

In summary, the present study demonstrates the circulation in France between 2017 and 2022 of new HCoV‐OC43 genotypes initially detected in China, and deciphered their dynamics over time. It contributes to start filling a huge gap in HCoV‐OC43 genome availability worldwide and in France, and points out the tremendous imbalance between the amounts of genomic data on SARS‐CoV‐2 and on another human coronavirus discovered decades earlier and known to cause frequent and sometimes severe infections in humans. A large proportion (77%) of the genomes analyzed here were obtained from samples collected during the 2020–2022 period, due to an intensive screening of respiratory viruses during the first months of the COVID‐19 pandemic. This uneven temporal distribution might have introduced a bias that could have affected the accuracy of the assessment of the HCoV‐OC43 evolutionary dynamics over the whole study period. It is worthy to note that novel HCoV‐OC43 genotypes have been recurrently reported [13, 16, 47, 50, 52], including some involved in severe and fatal cases [13, 53], and this suggests that new variants will likely emerge in the near future. Besides, previously unannotated open reading frames (ORFs) were recently reported in the HCoV‐OC43 genome, including a putative nested ORF as well as short upstream ORFs, which warrants investigating for various isolates belonging to circulating genotypes the viral transcriptional and translational landscapes [54]. Furthermore, studies incorporating clinical and epidemiological data would be useful to explore potential associations between viral genotypes or recombination events and factors such as disease severity, patient age, or seasonality. Finally, further studies are needed to gain a better knowledge of whether the different HCoV‐OC43 genotypes are associated with particular transmissibility, antigenicity, replicative capacity, or pathogenicity, and of the determinants of the rise and fall of their epidemics.

## Author Contributions

**Bernard La Scola and Philippe Colson:** conceived and designed the experiments. **All authors:** contributed materials, analysis tools. **Hikmat Houmadi, Céline Boschi, Marine Lefebvre, Lorlane Le Targa, Jacques Fantini, Bernard La Scola, Philippe Colson:** analyzed the data. **Hikmat Houmadi, Bernard La Scola, Marine Lefebvre, Jacques Fantini, and Philippe Colson:** writing – original draft preparation. **All authors:** writing – review and editing. All authors have read and agreed to the published version of the manuscript.

## Ethics Statement

The present study has been registered on the Health Data Access Portal of Marseille public and university hospitals (Assistance Publique‐Hôpitaux de Marseille [AP‐HM]) with No. PADS24‐190 and was approved by the Ethics and Scientific Committee of AP‐HM.

## Conflicts of Interest

L.L.T. is an employee of BioSellal (Dardilly, France). B.L.S. and P.C. are scientific advisors of BioSellal and Triber companies. The remaining authors declare no conflicts of interest. Funding sources had no role neither in the design and conduct of the study; in the collection, management, analysis, and interpretation of the data; nor in the preparation, review, and final approval of the manuscript.

## Supporting information

SM Houmadi LaScola Colson JMV Oct2025 RevvvDD UNmarked.

## Data Availability

The HCoV‐OC43 genomes analyzed in this study were submitted to the Genbank database (https://www.ncbi.nlm.nih.gov/genbank/) (GenBank Accession no. PV471628‐PV471812).
