# Supplementary material for: Five‐Year (2017–2022) Evolutionary Dynamics of Human Coronavirus OC43 in Southern France Based on Whole Genome Next‐Generation Sequencing
Source: J Med Virol. 2025 Dec 3;97(12):e70726. doi: 10.1002/jmv.70726 (PMC12673430; doi:10.1002/jmv.70726)
Supplement: Supplementary file 1 — SM Houmadi LaScola Colson JMV Oct2025 RevvvDD UNmarked. [file JMV-97-e70726-s001.docx]

**SUPPLEMENTARY MATERIAL**

**Supplementary Figures**

**Supplementary Figures S1.** Bootscan representations for putative HCoV-OC43 recombinants

**A.** HCoV-OC43-0059_Sep-2022


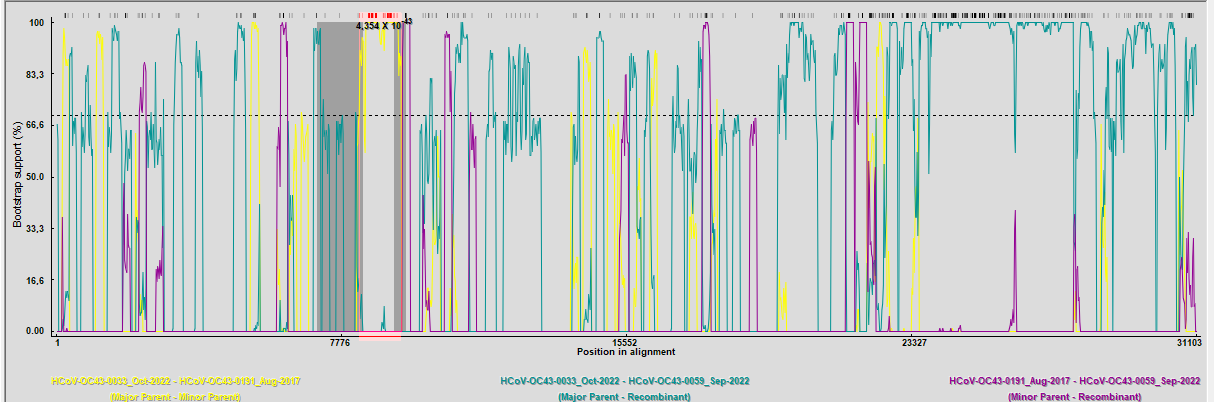


Putative genome recombinant: HCoV-OC43-0059_Sep-2022 Minor parental genome: HCoV-OC43-0191_Aug-2017 Major parental genome: HCoV-OC43-0033_Oct-2022

**B.** HCoV-OC43-0191_Aug-2017


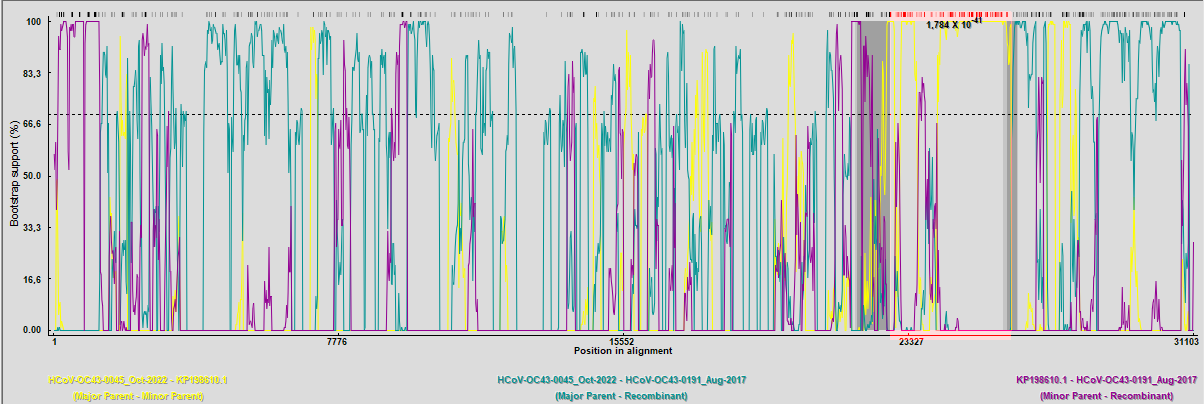


Putative genome recombinant: HCoV-OC43-0191_Aug-2017 Minor parental genome: KP198610.1 Major parental genome: HCoV-OC43-0045_Oct-2022

**C.** HCoV-OC43-0177_Sep-2018


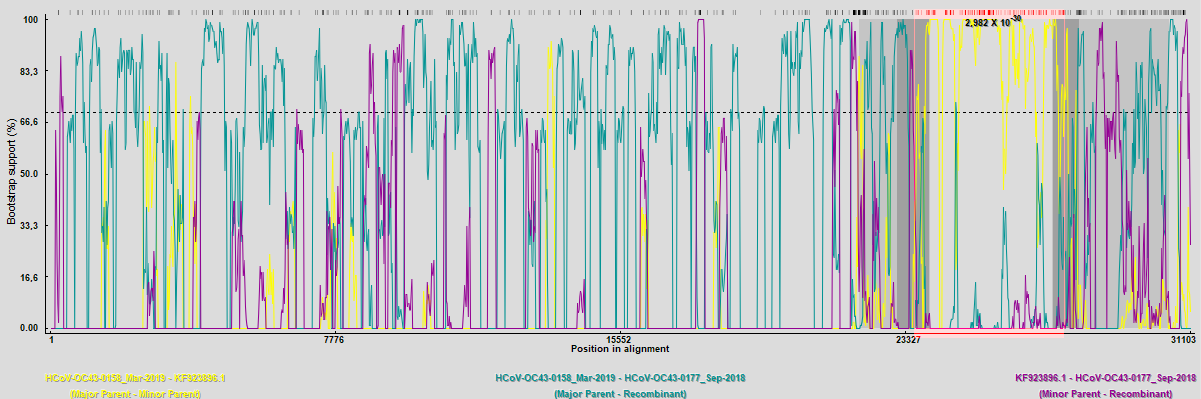


Putative genome recombinant: HCoV-OC43-0177_Sep-2018 Minor parental genome: KF923896.1 Major parental genome: HCoV-OC43-0158_Mar-2019

**D.** HCoV-OC43-0058_Sep-2022


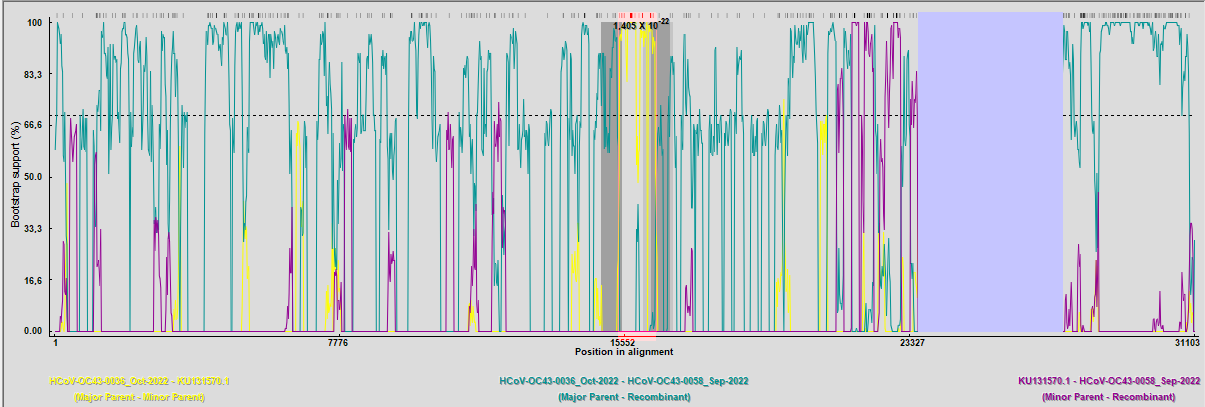


Putative genome recombinant: HCoV-OC43-0058_Sep-2022 Minor parental genome: KU131570.1 Major parental genome: HCoV-OC43-0036_Oct-2022

**E.** HCoV-OC43-0160_Nov-2018


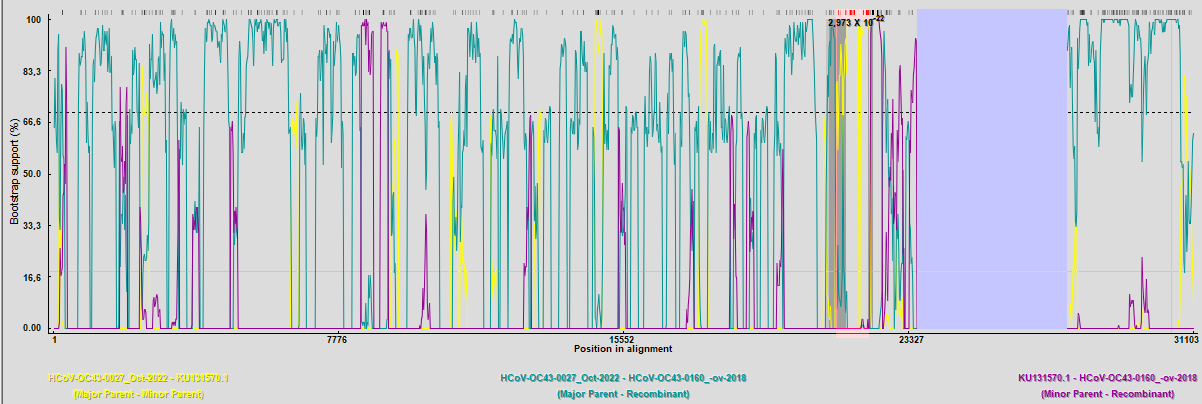


Putative genome recombinant: HCoV-OC43-0160_Nov-2018 Minor parental genome: KU131570.1 Major parental genome: HCoV-OC43-0027_Oct-2022

**Supplementary Figures S2.** Mixed effects model of evolution (MEME) applied to the different HCoV-OC43 genes

**A.** Mixed effects model of evolution (MEME) of Hemagglutinin esterase

**B.** Mixed effects model of evolution (MEME) of Spike


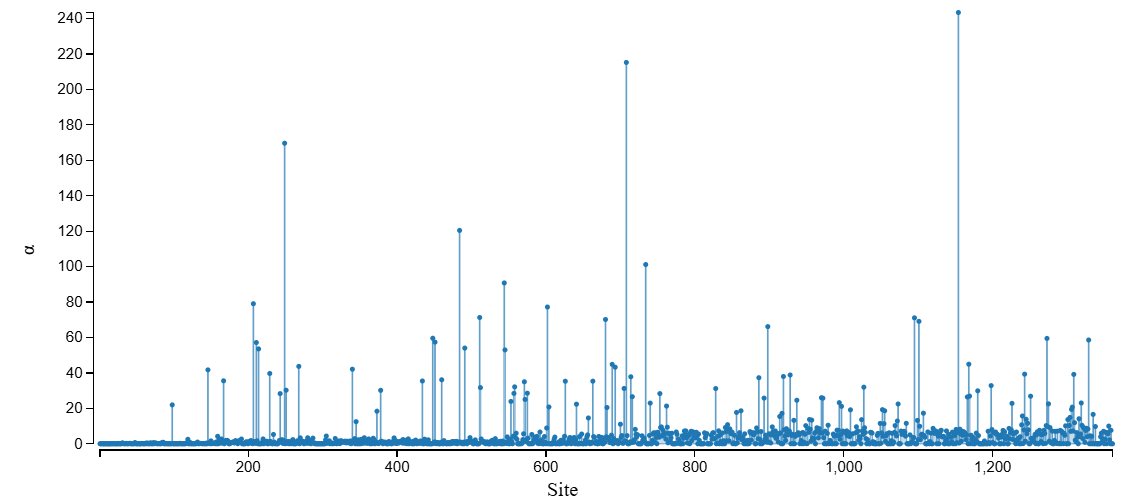


**C.** Mixed effects model of evolution (MEME) of Envelope


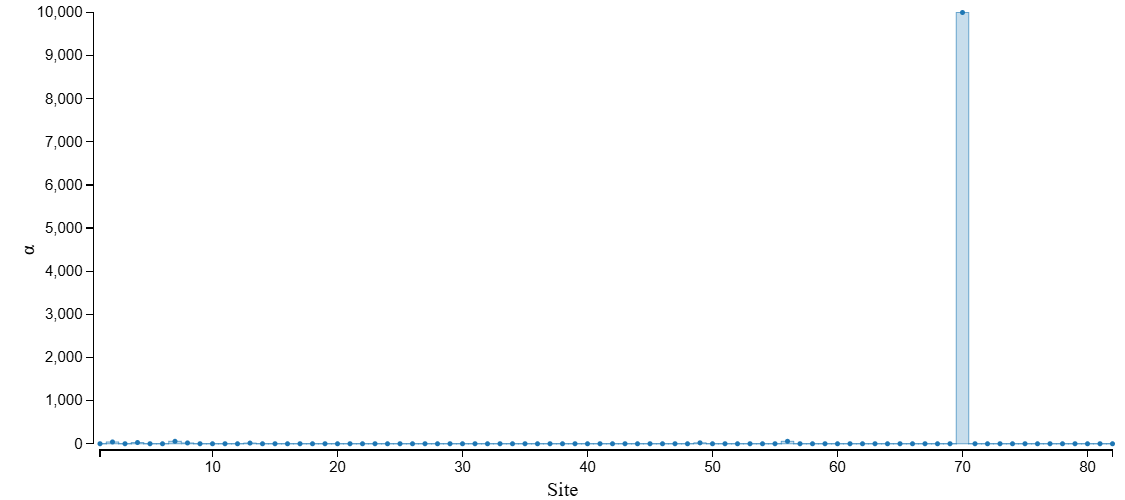


**D.** Mixed effects model of evolution (MEME) of Matrix


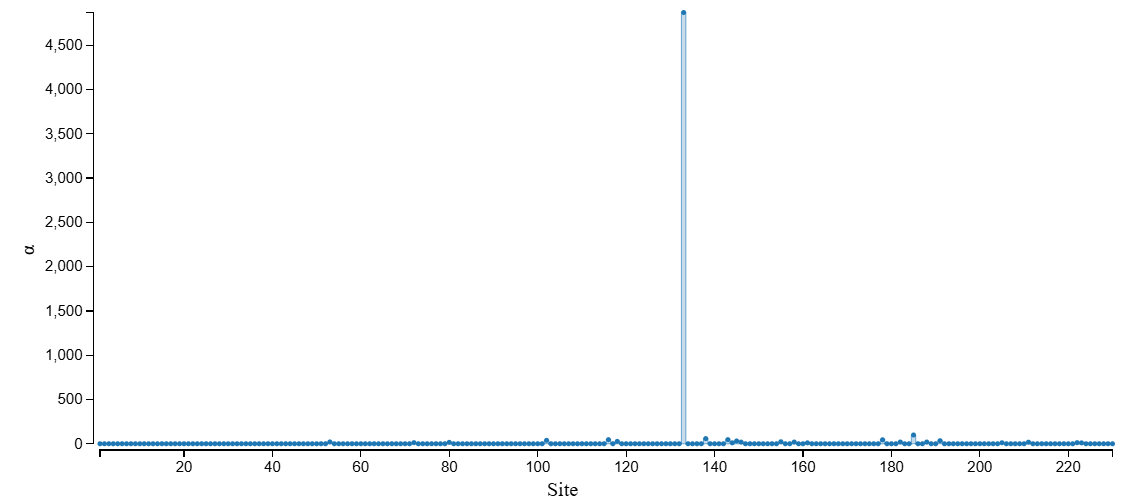


**E.** Mixed effects model of evolution (MEME) of Nucleocapsid


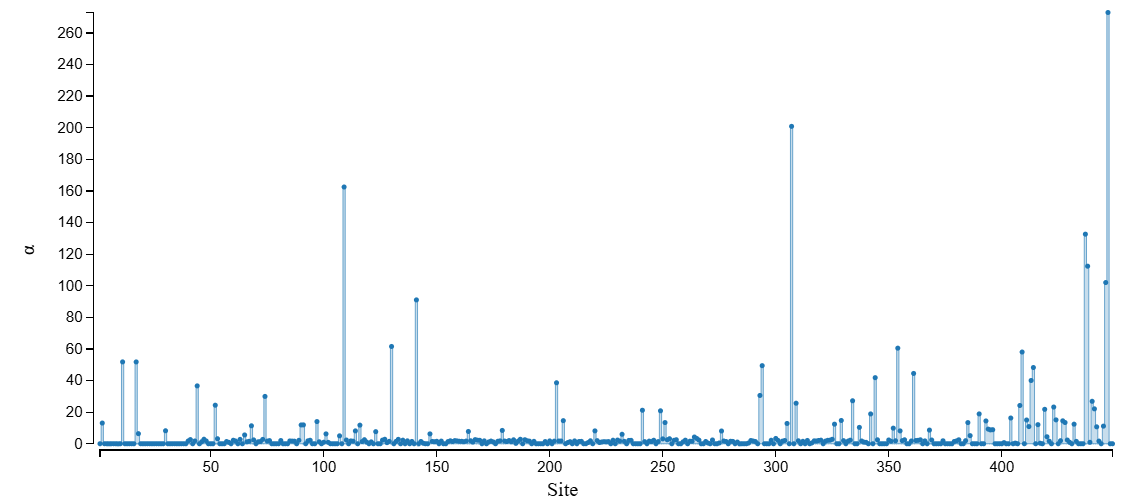


**F.** Mixed effects model of evolution (MEME) of Nsp3


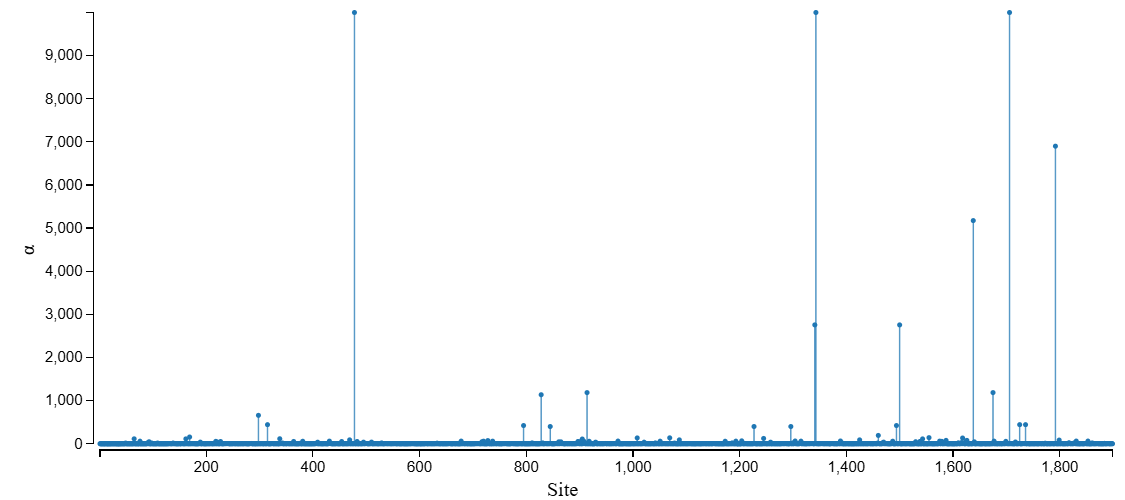


**G.** Mixed effects model of evolution (MEME) of Nsp5


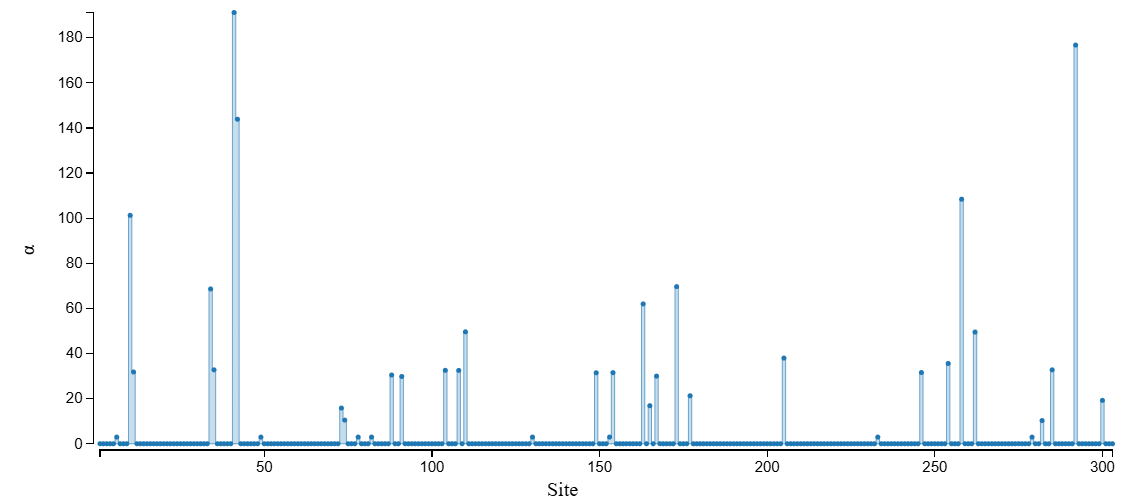


**H.** Mixed effects model of evolution (MEME) of Nsp7


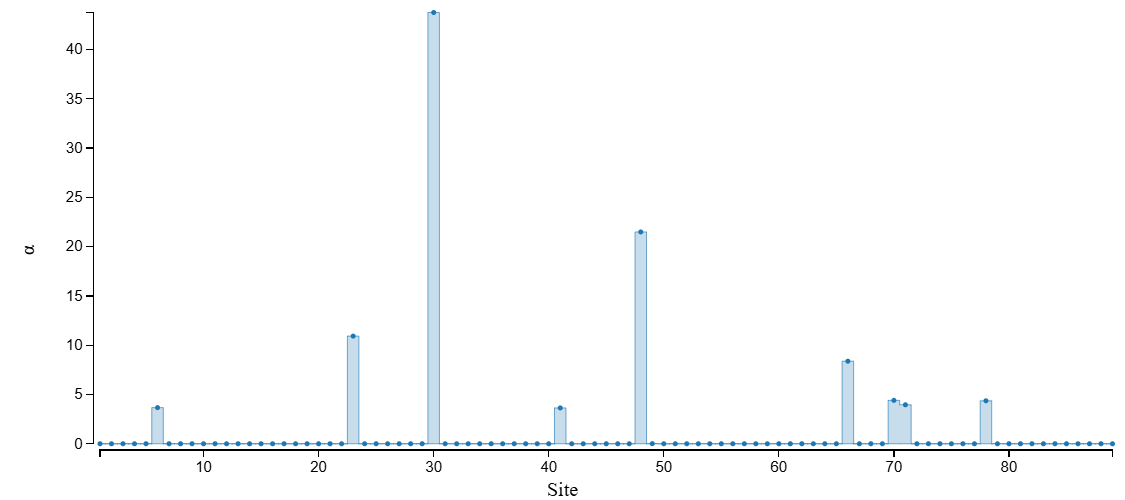


**I.** Mixed effects model of evolution (MEME) of Nsp8


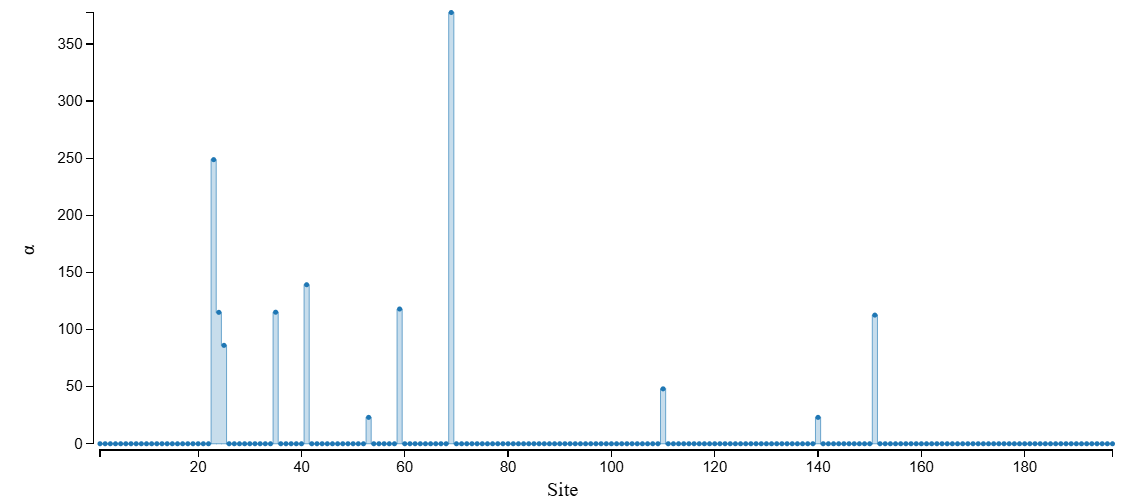


**J.** Mixed effects model of evolution (MEME) of Nsp10


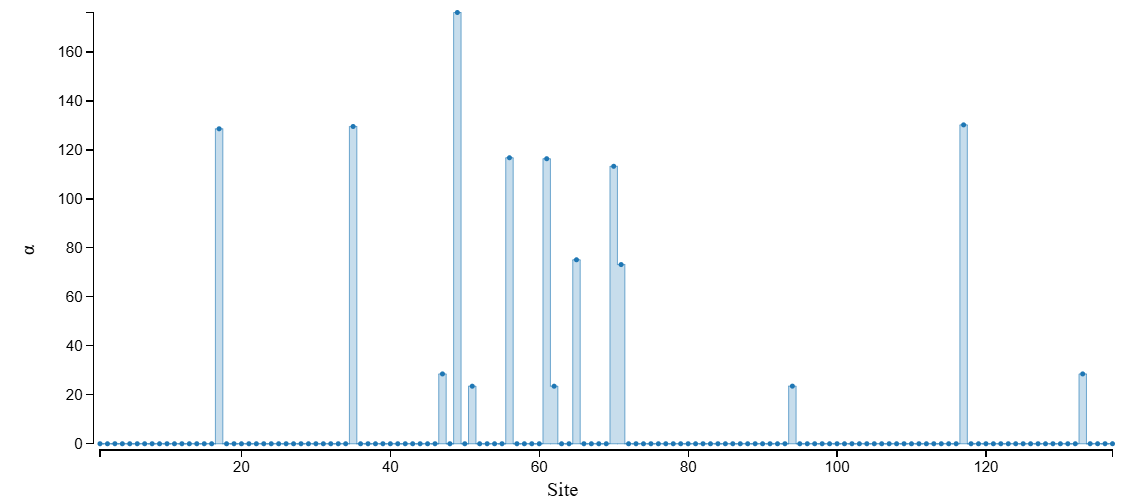


**K.** Mixed effects model of evolution (MEME) of Nsp12


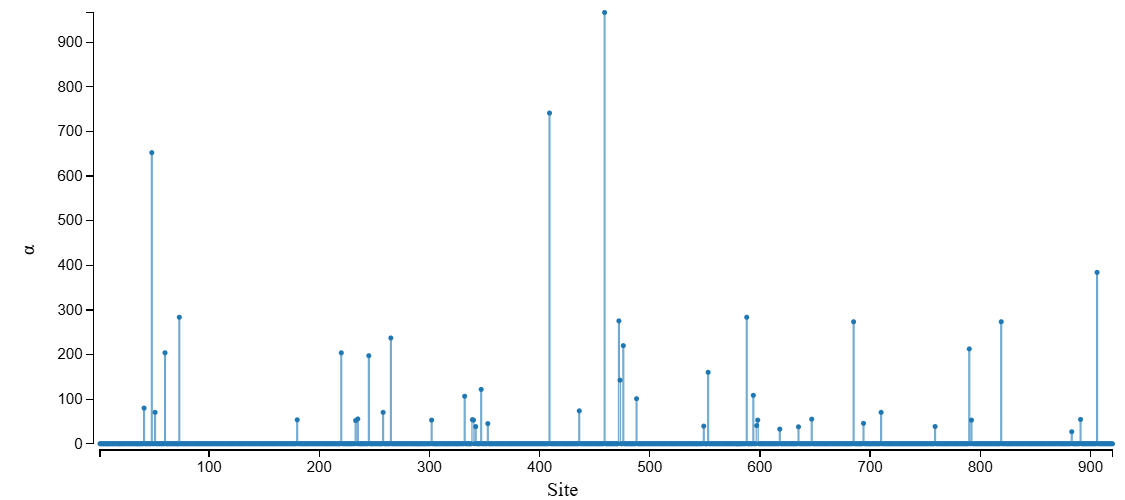


**L.** Mixed effects model of evolution (MEME) of Nsp13


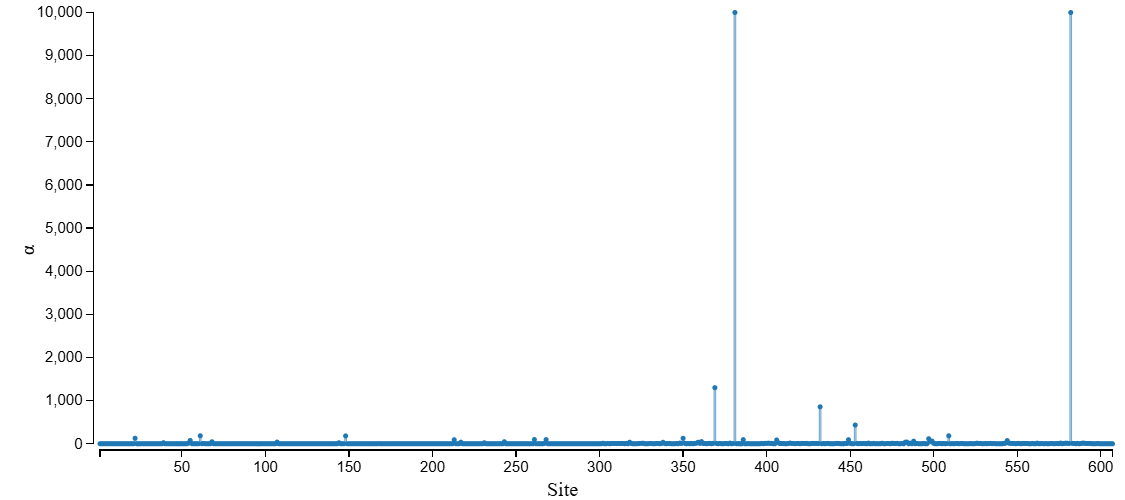


**M.** Mixed effects model of evolution (MEME) of Nsp14


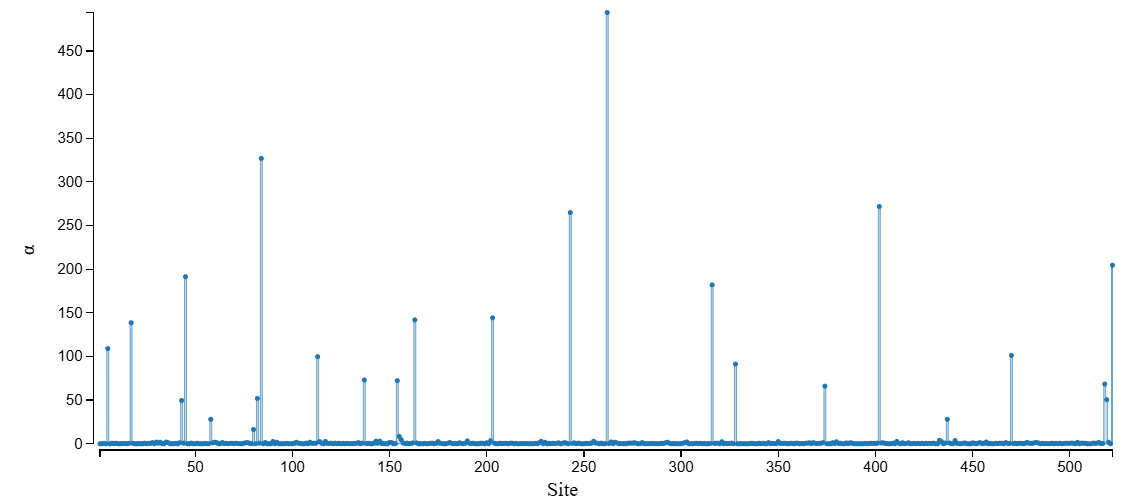


**N.** Mixed effects model of evolution (MEME) of Nsp15


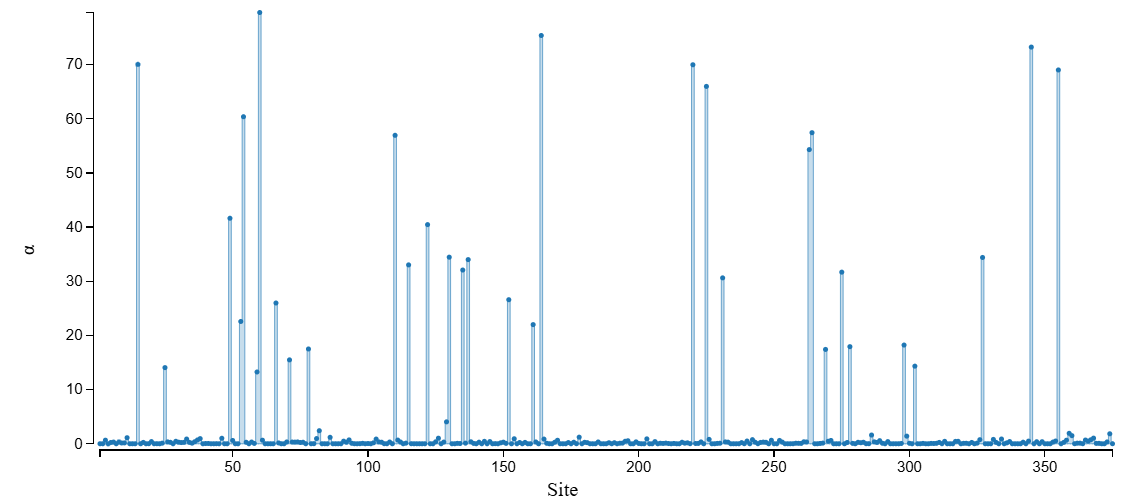


**O.** Mixed effects model of evolution (MEME) of Nsp16


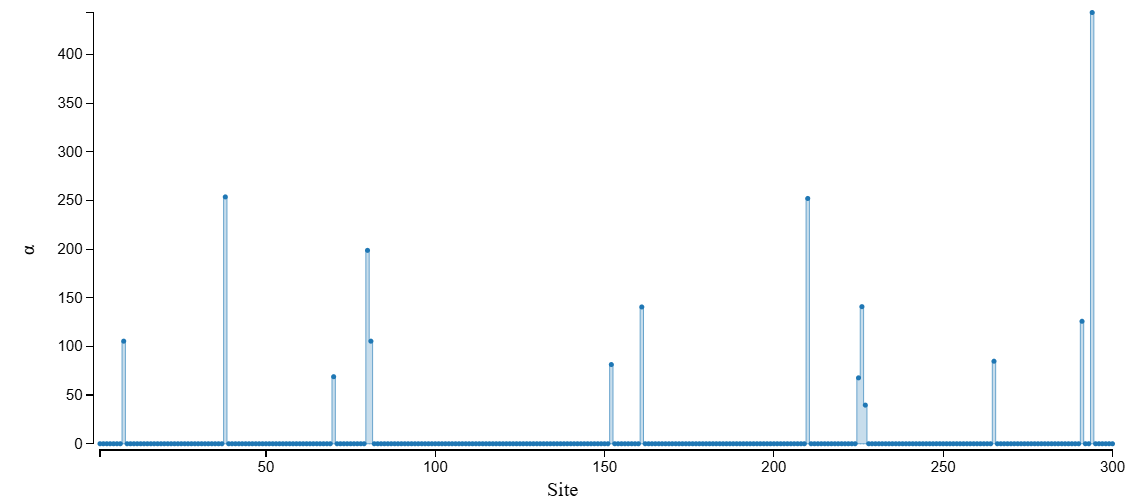


**Supplementary Tables**

**Supplementary Table S1.** Mixed Effects Model of Evolution (MEME) analysis

| Gene | Size (amino acids) | Positive/diversifying selection |
| --- | --- | --- |
| Hemagglutinin esterase | 1,284 | 109 |
| Spike | 4,077 | 119 |
| Matrix | 693 | 2 |
| Envelope | 255 | 0 |
| Nucleocapsid | 1,347 | 9 |
| Nsp3 | 5,697 | 20 |
| Nsp5 | 909 | 6 |
| Nsp7 | 267 | - |
| Nsp8 | 591 | - |
| Nsp10 | 411 | - |
| Nsp12 | 2,783 | 1 |
| Nsp13 | 1,809 | 9 |
| Nsp14 | 1,563 | 25 |
| Nsp15 | 1,125 | 17 |
| Nsp16 | 897 | 1 |

P value was significant when 0.1

**Supplementary Table S2.** Fixed Effects Likelihood (FEL) analysis

| Gene | Size (amino acids) | Number of sites under diversifying, positive selection | Number of sites under purifying, negative selection |
| --- | --- | --- | --- |
| Hemagglutinin esterase | 1,284 | 97 | 59 |
| Spike | 4,077 | 33 | 67 |
| Matrix | 693 | 0 | 6 |
| Envelope | 255 | 0 | 3 |
| Nucleocapsid | 1,347 | 0 | 41 |
| Nsp3 | 5,697 | 6 | 88 |
| Nsp5 | 909 | 3 | 21 |
| Nsp7 | 267 | - | 2 |
| Nsp8 | 591 | 0 | 4 |
| Nsp10 | 411 | 0 | 4 |
| Nsp12 | 2,783 | 1 | 25 |
| Nsp13 | 1,809 | 1 | 27 |
| Nsp14 | 1,563 | 50 | 25 |
| Nsp15 | 1,125 | 12 | 33 |
| Nsp16 | 897 | 0 | 12 |

P value was significant when <0.1
